# H727 cells are inherently resistant to the proteasome inhibitor carfilzomib, yet require proteasome activity for cell survival and growth

**DOI:** 10.1038/s41598-019-40635-1

**Published:** 2019-03-11

**Authors:** Min Jae Lee, Zachary Miller, Ji Eun Park, Deepak Bhattarai, Wooin Lee, Kyung Bo Kim

**Affiliations:** 1 Department of Pharmaceutical Sciences, University of Kentucky, Lexington, KY USA; 2 College of Pharmacy and Research Institute of Pharmaceutical Sciences, Seoul National University, Seoul, Korea

## Abstract

The second-in-class proteasome inhibitor (PI) carfilzomib (Kyprolis, Cfz) has contributed to a substantial advancement in multiple myeloma treatment by improving patient survival and quality of life. A considerable portion of patients however display intrinsic resistance to Cfz. Our mechanistic understanding of intrinsic Cfz resistance is limited due to a lack of suitable cell-based models. We report that H727 human bronchial carcinoid cells are inherently resistant to Cfz, yet susceptible to other PIs and inhibitors targeting upstream components of the ubiquitin-proteasome system (UPS). These results indicate that H727 cells remain dependent on the UPS for cell survival and growth despite harboring intrinsic resistance to Cfz. Alterations in the composition of proteasome catalytic subunits via interferon-γ treatment or siRNA knockdown results in sensitization of H727 cells to Cfz. We postulate that a potential link may exist between the composition of proteasome catalytic subunits and the cellular response to Cfz. Overall, H727 cells may serve as a useful cell-based model for *de novo* Cfz resistance and our results suggest previously unexplored mechanisms of *de novo* PI resistance.

## Introduction

The proteasome, an evolutionarily conserved multiprotease complex, is responsible for the controlled degradation of intracellular proteins. These include defective ribosomal products (DRiPs), oxidized proteins, and tightly-regulated cellular signaling proteins involved in cell cycle progression, immune response, apoptosis, signal transduction, and stress responses^1^. Proteins are targeted for proteasomal degradation by ubiquitination, a process involving a cascade of three enzymes: E1 (ubiquitin activating enzyme), E2 (ubiquitin conjugating enzyme), and E3 (ubiquitin ligase). Once protein substrates are polyubiquitinated, they are recognized by the proteasome’s regulatory particle (19S complex) and degraded within the central chamber of the core particle (20S complex) of the proteasome. The 20S proteasome core is composed of four stacked heptameric rings: two outer α-rings and two inner β-rings. In mammalian proteasomes, each β-ring harbors three catalytic β-subunits, β1, β2, and β5 which display different substrate preferences, respectively referred to as caspase-like (C-L), trypsin-like (T-L) and chymotrypsin-like (CT-L) activities. It was generally thought that 20S proteasomes exist in two main types, namely, the constitutive proteasome (cP) and the immunoproteasome (iP). Immunoproteasomes differ from cP by the replacement of β1, β2, and β5 with the homologous catalytic subunits β1i, β2i, and β5i. Interestingly, recent investigations revealed that certain tissues and some cancer cells carry non-standard types of 20S proteasomes (referred to as hybrid or intermediate proteasomes), which contain mixed assortments of cP and iP catalytic subunits, such as β1i-β2-β5i^2–6^. It was further reported that these non-standard proteasomes may confer differing sensitivities to proteasome inhibitors (PIs) as compared to cPs or iPs^4,5,7^, but the clinical implications of these non-standard proteasomes remain unknown.

The proteasome is an effective anticancer target, validated by the clinical success of the FDA approved proteasome inhibitors (PIs) bortezomib (Velcade, Btz), carfilzomib (Kyprolis, Cfz), and ixazomib (Ninlaro, Ixz) as multiple myeloma (MM) therapies. PIs have become an integral part of MM treatment and have contributed to a major uplift of patient outcomes over the past decade and a half. While the first-in-class PI drug Btz and the first oral PI Ixz utilize boronic acid pharmacophores, the second-generation PI Cfz harbors an epoxyketone that irreversibly inactivates the proteasome with high mechanistic selectivity^8,9^. This selectivity affords Cfz a reduction in off-target interactions yielding an improved safety profile over Btz, most notably a reduced incidence of severe peripheral neuropathy^10^. With positive results from recent phase III clinical trials^11–16^, Cfz is now firmly placed as a mainstay of refractory MM therapy. Nevertheless, a considerable portion of MM patients are refractory to Cfz or develop resistance after prolonged Cfz treatment. A meta-analysis of 14 clinical trials found that 44% of patients could not achieve a minimal response or better^17^. As a monotherapy in patients with relapsed MM, for example, the response rates for Cfz were in the ranges of 25–40%^18^. When used in combination with other drugs (often with dexamethasone and/or lenalidomide), response rates substantially improved, but a significant subset of non-responders persisted^16,19–22^. Even for those who initially respond to Cfz-based therapy, disease eventually relapses with a median progression-free-survival (PFS) of ~17–26 months^20,21^. To date, considerable efforts have been put forth toward the development of new therapeutics for these Cfz non-responders without significant progress. Efforts to tackle this problem have been significantly hampered by a limited understanding of the biological mechanisms underlying Cfz resistance.

Mechanistic investigations of Cfz resistance have so far utilized cancer cell lines adapted to gradually increasing concentrations of Cfz, revealing that the overexpression of P-glycoprotein (P-gp) and mutations or amplification/overexpression of proteasome catalytic subunits are largely responsible for acquired Cfz resistance observed in established cell lines^23–25^. To date, cell-based models of *de novo* Cfz resistance are unavailable. Here, we report for the first time that H727 cells (derived from a human bronchial carcinoid tumor) are inherently resistant to Cfz, yet remain dependent on the proteasome for their survival and growth. Our current results suggest that *de novo* Cfz resistance observed in H727 cells may be mediated at the 20S proteasome level, providing previously unknown insights into the mechanisms of *de novo* PI resistance.

## Results

### H727 cells are inherently resistant to Cfz

**Figure 1:**
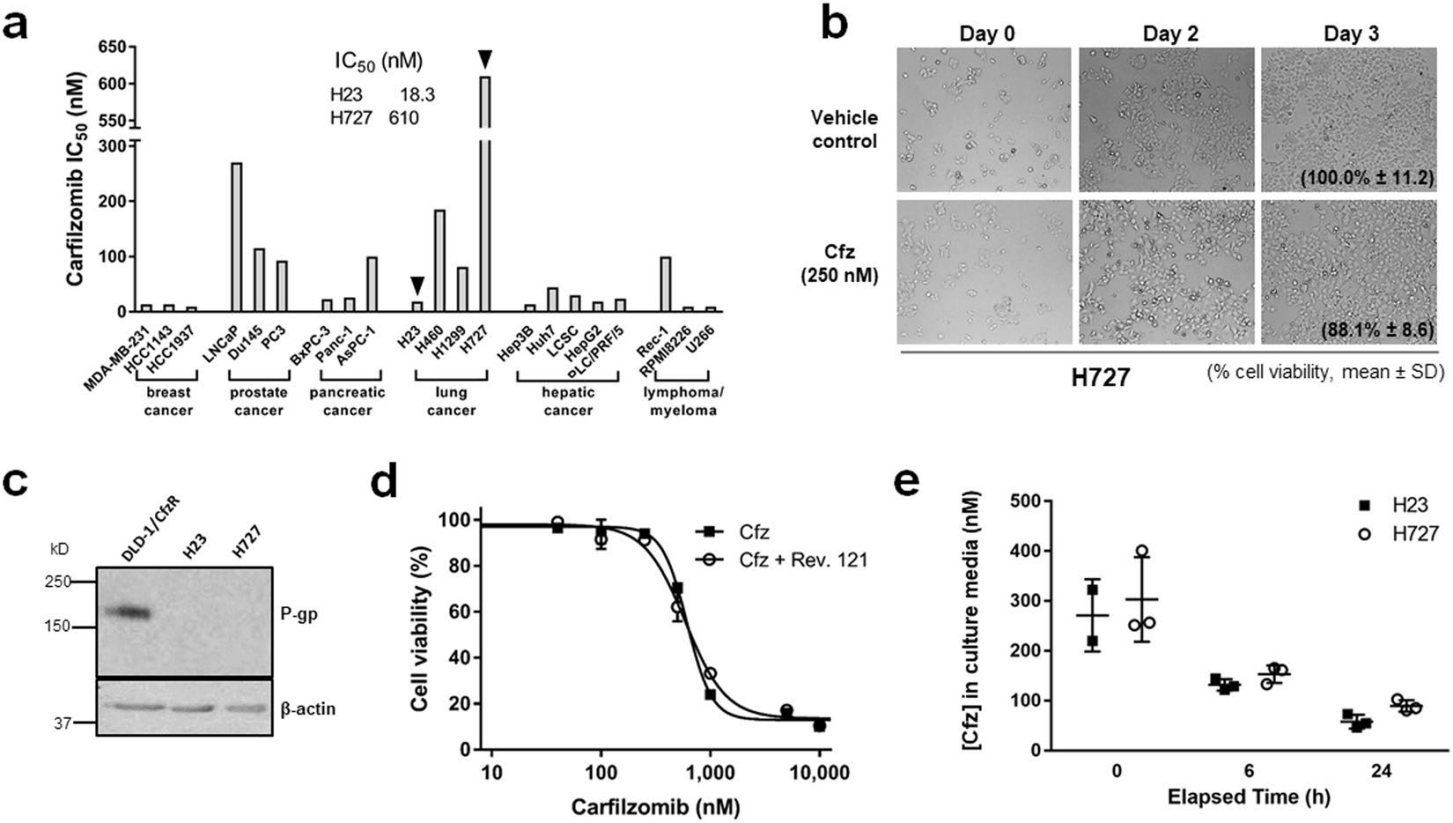
(**a**) Cell viability (IC_50_ values) for a panel of established cancer cell lines as measured by MTS assay following incubation with carfilzomib (Cfz) for 72 h. H727 cells are most resistant to Cfz among 21 tested cell lines. (**b**) Representative images of H727 cells growing in the presence of Cfz (250 nM) assessed via light microscopy. On day 3 of Cfz treatment, live cell not stained with trypan blue were counted (expressed as % cell viability relative to the vehicle control; no statistically significant difference between Cfz-treated H727 cells and the vehicle control by Student’s t-test, P value > 0.1, n = 3). (**c**) Immunoblotting results showing no detectable expression of P-glycoprotein (P-gp) in H727 cells. DLD-1 cells with acquired Cfz resistance via P-gp upregulation (DLD-1/CfzR) were used as a positive control. (**d**) The co-treatment of reversin-121 (7.5 µM, P-gp inhibitor) did not affect the sensitivity of H727 cells to Cfz. The IC_50_ values did not show statistically significant difference between in the presence and absence of reversin-121 (Student’s t-test). (**e**) The levels of remaining Cfz in culture media were comparable between H727 and H23 cells (no statistically significant differences, t-tests the Holm-Sidak method to correct for multiple comparisons with α = 0.05).

When the IC_50_ values for Cfz were assessed in a panel of 21 human cancer cell lines derived from various types of cancer, H727 lung cancer cells were most resistant to Cfz, with a IC_50_ value of 610 nM (Fig. 1a). Even in the presence of 250 nM of Cfz which usually induces >95% loss of viability in other cancer cell lines, H727 cells survived and grew normally (Fig. 1b; the relative cell viability on day 3 was 88.1% based on the counting of cells not stained with trypan blue dye (Sigma-Aldrich, St. Louis, MO)). While the mechanisms of intrinsic resistance to Cfz has not been reported to date, several studies have shown that P-gp can contribute to acquired resistance to Cfz observed in cancer cell line models and clinical samples from patients with prior Cfz therapy^24,26,27^. To test whether P-gp plays a role in the *de novo* Cfz resistance of H727 cells, we performed immunoblotting analysis but found no detectable P-gp expression (Fig. 1c). Furthermore, treatment of H727 cells with reversin 121, a dipeptide P-gp inhibitor, did not significantly impact the IC_50_ value of Cfz (Fig. 1d), confirming a P-gp-independent mechanism of resistance. Direct sequencing analyses also indicated that the *PSMB5* (encoding β5) and *PSMB8* (encoding β5i) genes in H727 cells harbor no mutations (Supplementary Fig. S1). Next, we examined the possibility that Cfz may undergo rapid metabolic inactivation in H727 cells. We treated H727 and H23 (Cfz-sensitive) cells with 500 nM Cfz and collected culture media to measure the levels of remaining Cfz at 6 or 24 h post-treatment. The level of remaining drug was overall comparable between H727 and H23 cells although a slight difference was noted at 24 h (Fig. 1e). We attempted to compare the intracellular drug levels by quantifying the remaining drug levels in lysates of H727 and H23 cells, but the levels were below the lower limit of quantitation (<5 nM) of our current analytical assay. Although it was not feasible to assess the intracellular drug levels, H727 cells contained Cfz in the culture media at the level comparable to or slightly higher than H23 cells. Assuming that Cfz primarily enters cells via passive diffusion (no report yet supporting the presence of uptake transporters for Cfz as far as we know), it appears unlikely that H727 cells have intracellular Cfz levels much lower than H23 cells. Taken together, *de novo* resistance of H727 cells was not explained by previously reported mechanisms such as P-gp upregulation, genetic mutations in proteasome catalytic subunits, or enhanced metabolic inactivation of Cfz.

### The UPS/proteasome remains essential for the survival of H727 cells

**Figure 2:**
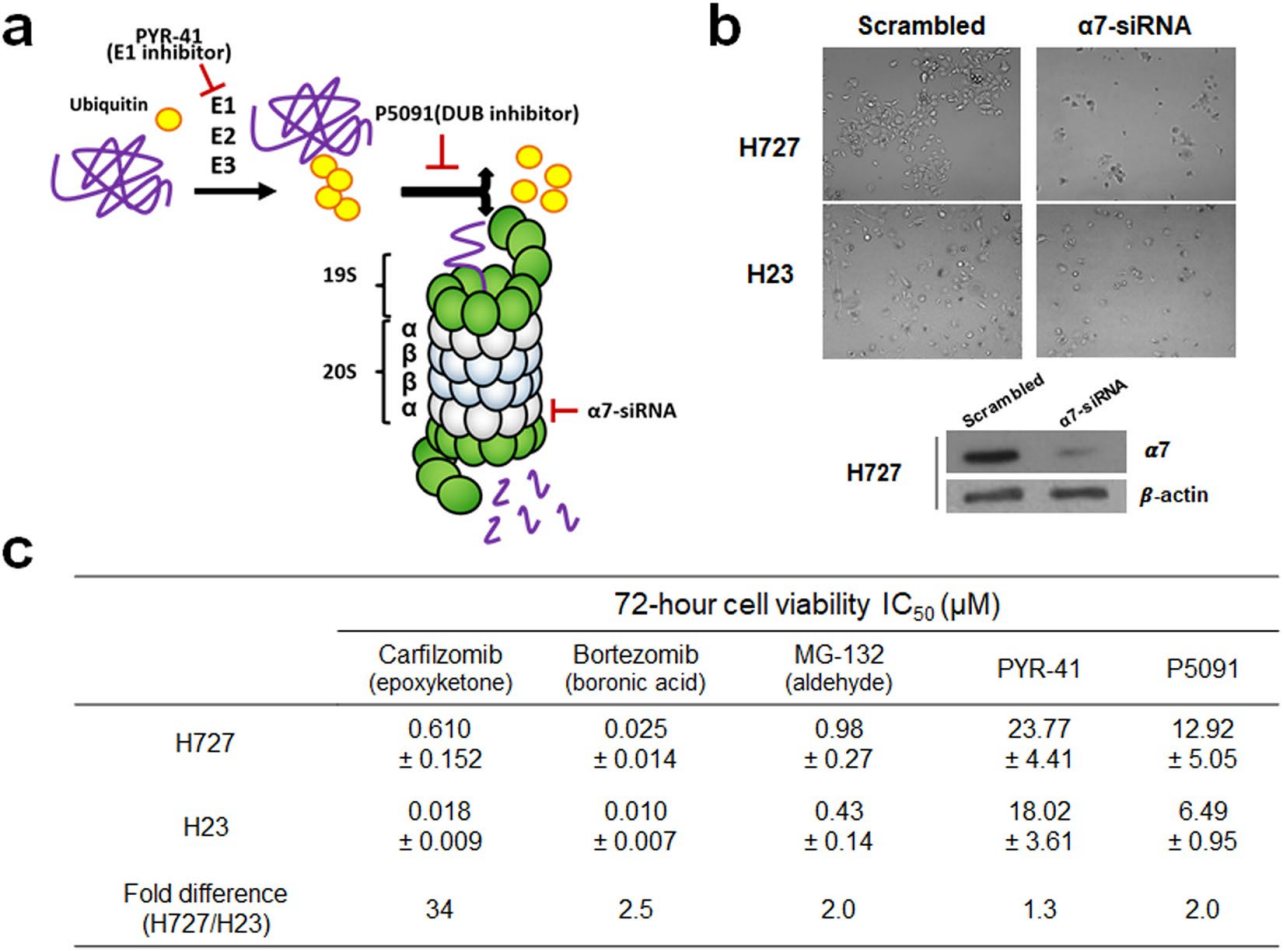
(**a**) Diagram showing the ubiquitin-proteasome system (UPS) and inhibitors targeting upstream components of the proteasome. (**b**) Knockdown of proteasome α7 subunit in H727 cells effectively induced cell death to a similar extent as observed in H23 cells (images taken 48 h post-transfection). Immunoblotting analysis was performed to verify the efficient knockdown of α7 in H727 cells. (**c**) Comparison of the sensitivity (IC_50_ values) of H727 and H23 cells to carfilzomib, bortezomib, MG-132, PYR-41 (an E1 inhibitor) and P5091 (an USP7/USP47 inhibitor). Data are shown as mean ± SD derived from a single non-linear regression based on n = 3–4 replicates per compound per concentration.

Cfz selectively inhibits the proteasome by covalently modifying the N-terminal catalytic threonine residues of 20S proteasome catalytic subunits at nanomolar concentrations while not inhibiting other proteases at concentrations as high as 50 µM^8,28,29^. We thus considered whether H727 cells might have adaptations to endure reduced levels of 20S proteasome function, for example, the use of non-proteasomal protein degradation pathways thus reducing proteasome load. In order to determine whether the 20S proteasome is essential for the survival and growth of H727 cells as it is in Cfz-sensitive cell lines such as H23, we first transfected cells with siRNAs targeting the proteasome α7 subunit to block 20S proteasome assembly (Fig. 2a,b)^30,31^. After 3 days post-transfection, we observed nearly complete cell death for both H727 and H23 cells (Fig. 2b). The extent and time-course of cell death incurred by α7 knockdown was comparable between H727 and H23 cells, indicating that H727 cells are highly dependent on the 20S proteasome. These results suggested that H727 cells may still respond to proteasome inhibitors other than Cfz. In order to examine this, we treated H727 cells with alternative PIs, particularly ones with differing pharmacophores or structures, such as Btz (a peptide boronic acid) and MG-132 (a peptide aldehyde). These PIs were indeed highly effective in killing H727 cells and their IC_50_ values were comparable between H727 and H23 cells (Fig. 2c). We also used two inhibitors targeting non-proteasomal UPS components: PYR-41, an inhibitor of ubiquitin E1 ligase and several DUBs, and P5091, a specific USP7/USP47 inhibitor^32^. Both PYR-41 and P5091 were cytotoxic in H727 and H23 cells with comparable potencies (Fig. 2c). These results further support that H727 cells remain dependent on the ubiquitin-proteasome system, despite their *de novo* resistance to Cfz.

### H727 cells have a distinct composition of proteasome catalytic subunits

**Figure 3:**
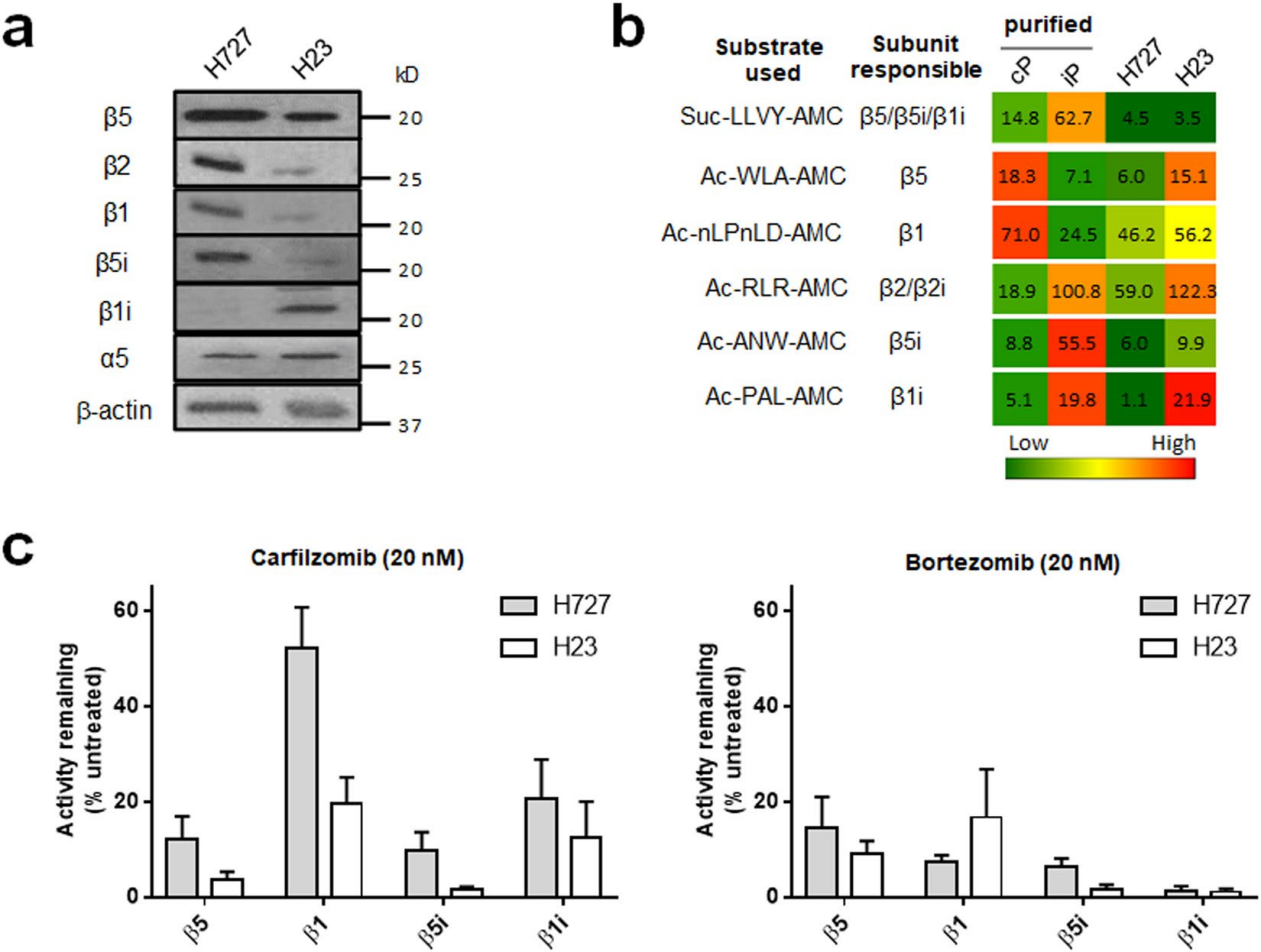
(**a**) Immunoblots showing the expression of cP and iP catalytic subunits in H727 and H23 cells. Immunoblots for additional cell lines displaying differential expression of proteasome catalytic subunits are presented in Supplementary Fig. S2. (**b**) Heat map displaying differential proteasome activity profiles in H727 and H23 cell lines. Purified human 20S cP and iP were used as controls for individual subunits: 20S cP for β5 and β1 and 20S iP for β5i and β1i. The numbers represent hydrolysis rates of respective substrates (RFU/min, mean values derived from three technical replicates) and were converted to color format and clustered by using the program “R” (http://www.R-project.org). (**c**) Remaining catalytic activities of individual proteasome subunits in H727 and H23 cells 4 h after treatment with 20 nM of carfilzomib (left panel) or 20 nM bortezomib (right panel). Data are presented as mean ± SD derived from three technical replicates.

To account for the sensitivity of H727 cells to other PIs, we hypothesized whether the subunit composition at the 20S proteasome level may contribute to *de novo* resistance of H727 cells to Cfz. To test this hypothesis, we compared the proteasome catalytic subunit expression and activity profiles of H727 and H23 cells via immunoblotting analysis and kinetics assays using fluorogenic substrates for individual subunits (β1, β5, β1i, β5i). In the case of the β2 and β2i subunits, their combined trypsin-like activity was assessed due to the lack of a specific fluorogenic substrate that can distinguish the two subunits. As shown in Fig. 3a, the expression pattern of proteasome catalytic subunits in H727 cells differed from that in H23 cells. H727 cells expressed high levels of β1, β2, and β5i, while β1i expression was undetectable. The expression profile of catalytic subunits in H727 cells was not consistent with those typically expected for the two main 20S proteasome subtypes, namely a set of β1-β2-β5 for cP or an immuno-subunit set of β1i-β2i-β5i for iP. Substantial differences were also noted when the activity profiles of proteasome catalytic subunits were compared between these two cell lines using subunit-selective fluorogenic substrates (Fig. 3b, the relative hydrolysis rates for each probe substrate shown as a heat map and numbers)^33^. Interestingly, the activity profiles of individual catalytic subunits showed discrepancies with the protein levels of the respective catalytic subunits. We suspect that the observed differences may reflect the complex relationship between proteasome structure and function (e.g. contributions of post-translational modifications, regulatory particles, or non-standard composition of proteasome catalytic subunits to the hydrolysis rates of fluorogenic substrates).

We next examined whether individual subunits of proteasomes in H727 cells may display different proteasome inhibition profiles than those of H23 cells. We treated H727 and H23 cells with 20 nM of Cfz for 4 h and measured the remaining activities of individual catalytic subunits relative to vehicle-treated control cells. As shown in Fig. 3c (left panel), more than 80% of β5, β5i and β1i activities were blocked by Cfz for both H727 and H23 cells. On the contrary, over 50% of β1 activity persisted in H727 cells, but not in H23 cells (Fig. 3c, left panel). It remains unclear whether the remaining β1 activity contributes to *de novo* resistance of H727 cells to Cfz. On the other hand, Btz treatment resulted in over 80% inhibition across all catalytic subunits in both H727 and H23 cell lines, which may explain the high sensitivity of both cell lines to Btz (Fig. 3c, right panel). These results support that the 20S proteasomes present in H727 cells may be functionally different from those in Cfz-sensitive H23 cells.

### Upregulation of β1i and β5i levels by IFN-γ sensitizes H727 cells to Cfz

**Figure 4:**
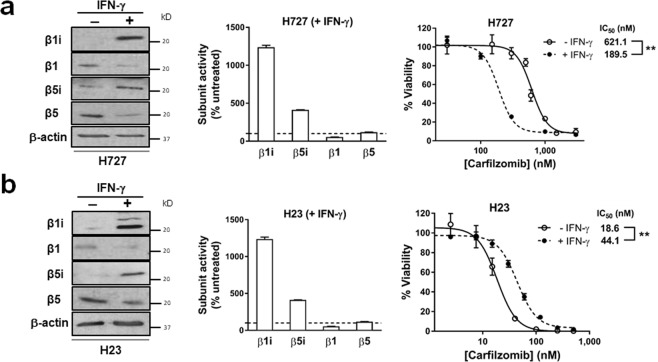
Effect of IFN-γ pretreatment on carfilzomib sensitivity of H727 (**a**) and H23 (**b**) cells. IFN-γ (150 U⋅ml^−1^) pretreatment for 24 h led to increased expression (left panel) and activity (middle panel) of proteasome immuno-subunits, and sensitized H727 cells to Cfz, while desensitizing H23 cells towards Cfz. The IC_50_ values displayed a statistically significant difference between IFN-γ-pretreated cells and vehicle control (P value < 0.01, n = 3, Student’s t-test comparing the log transformed IC_50_ values obtained from three independent runs).

Based on the differential expression pattern of proteasome catalytic subunits in H727 as compared to H23, we hypothesized that the composition of proteasome catalytic subunits may impact the sensitivity of H727 cells to Cfz. To test this hypothesis, we altered catalytic subunit composition in H727 using interferon-γ (IFN-γ) treatment. IFN-γ’s ability to upregulate immuno-subunits (β1i, β2i, and β5i) and to induce IP formation has been well-documented^34–38^. As shown in Fig. 4a, incubation of H727 cells with IFN-γ (150 U⋅ml^−1^) 24 h prior to Cfz treatment resulted in upregulation of immuno-subunit expression and corresponding increases in their activity. IFN-γ pre-treatment caused a significant decrease in Cfz IC_50_ values from 621.1 to 189.5 nM in H727 cells. When H23 cells were pre-treated with IFN-γ, the Cfz IC_50_ values changed in the opposite direction (IC_50_ values increased from 18.6 to 44.1 nM, Fig. 4b). With IFN-γ pre-treatment, the fold differences in IC_50_ values between the two cell lines were reduced from 33-fold to 4.2-fold.

### Alteration of proteasome catalytic subunit composition affects H727 Cfz sensitivity

**Figure 5:**
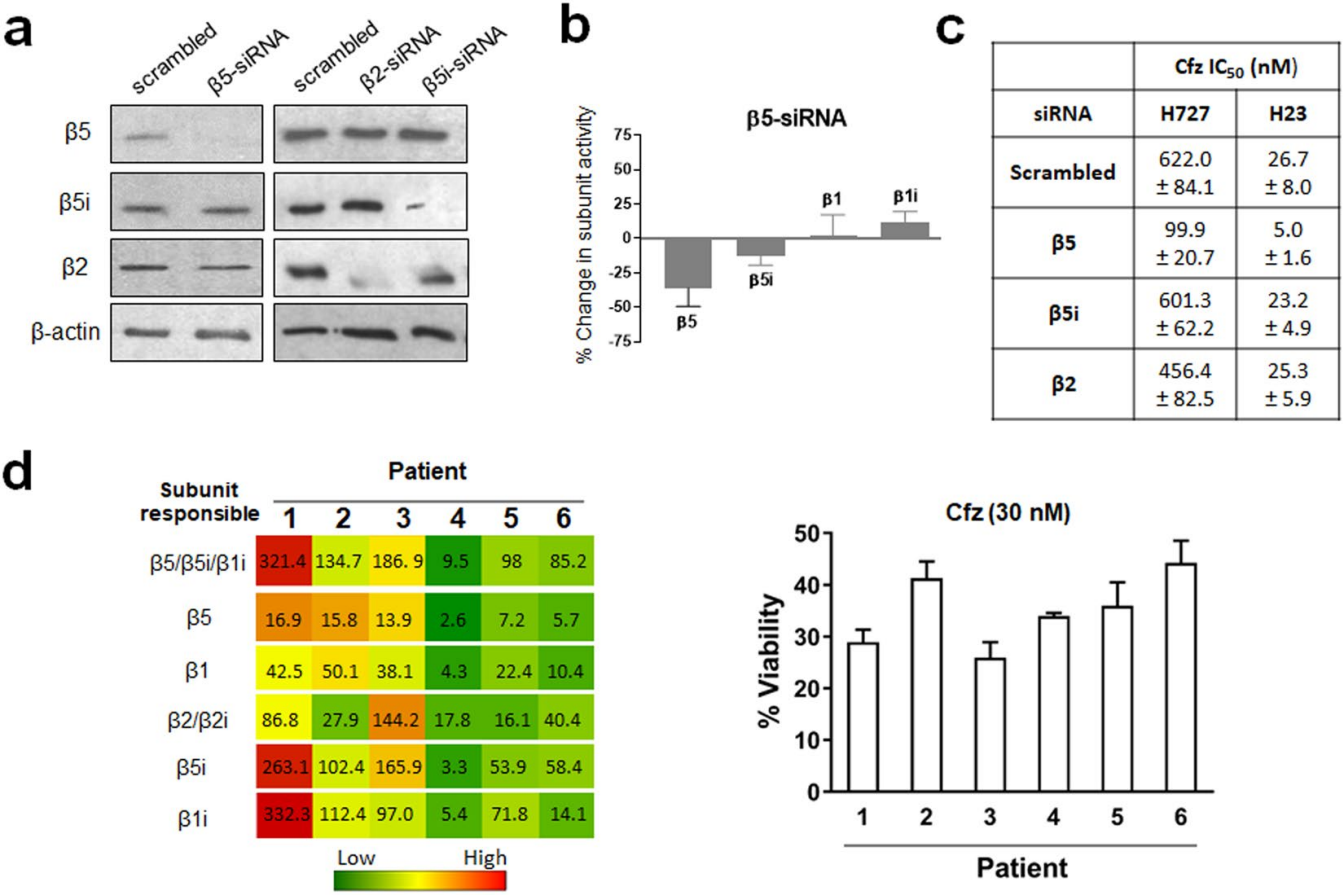
(**a**) Immunoblots of proteasome catalytic subunits in H727 cells transfected with siRNA targeting β5, β2 or β5i. (**b**) The catalytic activity of β5 subunit was decreased in H727 cells transfected with siRNA targeting β5 compared with H727 cells transfected with scrambled siRNA. (**c**) Effects of siRNA knockdown of β5, β5i, or β2 on Cfz sensitivity (IC_50_ values) in H23 and H727 cells. Data are shown as mean ± SD derived from a single non-linear regression based on n = 3 replicates per compound per concentration. (**d**) Heat map showing proteasome catalytic subunit activity profiles of 6 PI-naïve patient MM samples purchased from Conversant Biologics and AllCells (left). The numbers represent hydrolysis rates of respective substrates (RFU/min, mean values derived from three technical replicates) and were converted to color format and clustered by using the program “R” (http://www.R-project.org) (left panel). Carfilzomib (Cfz) cell viability of the same 6 patient MM cells was measured via CellTiter-Glo Luminescent Cell Viability Assay (right panel).

In order to further investigate a causal relationship between the composition of proteasome catalytic subunits and Cfz sensitivity, we sought to alter the composition of proteasome catalytic subunits in H727 cells in a more selective manner using an siRNA pool targeting the abundantly expressed β5 subunit. We expected that β5i will substitute for β5 during proteasome assembly, forming 20S complexes with altered catalytic subunit composition. When β5 was knocked down (verified via immunoblotting and activity assays, Fig. 5a,b), H727 cells grew normally with modest upregulation of β5i. Despite their normal growth, H727 cells were significantly sensitized to Cfz by β5 knockdown, shifting the IC_50_ value from 622 to 99.9 nM (Fig. 5c). In contrast, the IC_50_ for Btz was only modestly affected, decreasing from 26 to 12 nM (Supplementary Fig. S3). Knockdown of other catalytic subunits such as β5i and β2 resulted in minimal changes in the IC_50_ values for Cfz. A similar pattern was observed in H23 cells where knockdown of β5i and β2 had little effect on Cfz sensitivity but β5 knockdown triggered a five-fold reduction in Cfz IC_50_ from 26.7 to 5.0 nM (Fig. 5c).

### Inter-patient variability in the proteasome activity profiles of primary MM cells is high

In order to assess whether the observed variability in proteasome activity profiles in PI-naïve cell line models reflects the Cfz sensitivity of clinical MM samples, we examined the proteasome activity profiles and degree of Cfz sensitivity using 6 MM samples from patients who have received no prior PI therapy. Similar to the results obtained using cell line models, the clinical samples also showed considerable variability in catalytic subunit activity profiles and Cfz sensitivity (Fig. 5d). Due to the limited sample quantities, we were not able to perform any further investigations on these samples. Based on these initial assessment, we cautiously speculate that differential Cfz sensitivity in these patient samples may be influenced by variability in proteasome catalytic subunit composition, perhaps partially accounting for the varied responses to Cfz observed in clinical trial results^11,15,20^.

## Discussion

The advent of Cfz therapy has improved outcomes for many MM patients. However, like many other anticancer agents, the successful clinical use of Cfz is significantly challenged by the presence of intrinsic and acquired drug resistance. A significant fraction of MM patients previously treated with Btz-containing regimens do not respond to Cfz and even those who initially respond to Cfz almost invariably develop resistance in the course of their treatment^11,17,39^. In order to design new and effective therapeutic strategies which avoid, eliminate, or bypass resistance, it is important to better understand the mechanisms of Cfz resistance. To date, investigations of Cfz resistance have largely focused on acquired resistance due to the relative ease of generating Cfz-adapted cancer cell lines and the availability of clinical samples derived from patients who have developed resistance after prolonged Cfz therapy. On the other hand, mechanistic investigations of *de novo* Cfz resistance have been scarce, due to the lack of appropriate cell line models and patient samples. In the current study, we report that H727 cells are intrinsically resistant to Cfz, potentially serving as a useful model for mechanistic investigations of *de novo* Cfz resistance. Given that H727 cells were sensitive to inhibitors of non-proteasomal targets in the UPS and PIs other than Cfz, we surmise that H727 cells harbor functionally active proteasomes and that complete or near-complete inhibition of proteasome catalytic activity is incompatible with survival in these cells. Based on our current results, a shift towards non-UPS protein degradation pathways appears unlikely since H727 cells remain highly sensitive to the inhibition of UPS components including the proteasome itself.

Despite their similar degrees of dependence on the proteasome or UPS for survival and growth, H727 and H23 cells respond differently to Cfz, to a degree of 33-fold difference in IC_50_ values. This may be in part due to cell line-dependent cell growth rates or genetic/molecular differences. However, the high sensitivity of H727 cells to other PIs suggests that Cfz resistance in H727 cells may be mediated at the 20S proteasome level. It has been reported that proteasome inhibitor resistance is often associated with increased levels of proteasome subunit catalytic activity, especially in models of acquired bortezomib resistance^40,41^. However, H727 cells displayed substantially low activities of individual catalytic subunits as compared to H23 cells. At present, it is unclear whether the low proteasome activities in H727 cells are involved in conferring Cfz resistance. Previously it was reported that 20S proteasomes harboring a mixed assortment of cP and iP catalytic subunits exist in cancer cells and that their PI sensitivity differs from those of standard cP or iP^5^. Our results also indicated not only differing expression levels of proteasome catalytic subunits between H727 and H23 cells, but also differing levels of subunit catalytic activity. These findings are consistent with the presence of non-standard 20S proteasome subtypes (other than cP and iP). Determination of the 20S proteasome subtypes present in H727 cancer cells may shed further light on the underlying mechanisms of *de novo* Cfz resistance in H727 cells. Determination of the subunit composition of intact 20S proteasomes in cells is challenging and several groups including ours are currently trying to develop bi-functional or fluorescent probes to facilitate these efforts^33,42,43^.

In summary, we show here that H727 cancer cells are intrinsically resistant to Cfz but remain dependent on the proteasome for their survival and growth. Current mechanistic studies suggest that the composition of proteasome catalytic subunits may play an important role in determining sensitivity to Cfz. Our study also implies that proteasome inhibition by alternative PIs may still be a valid therapeutic strategy for patients with relapsed MM after having received treatment with Cfz.

## Materials and Methods

### Cell lines and chemicals

MDA-MB-231, HCC1143, and HCC1937 breast cancer cells were purchased from the Korean Cell Line Bank (Seoul, Korea). Hep3B, Huh7, LCSC, HepG2, and PLC/PRF/5 hepatic cancer cells were a kind gift of Dr. Roberto Gedaly (College of Medicine, University of Kentucky). All other established cell lines were obtained from the American Type Culture Collection (ATCC, Rockville, MD). All cells were cultured according to the manufacturer’s protocol in 5% CO_2_ in medium. Cultured cell lines were tested for *Mycoplasma* contamination routinely every 6 months. Specifically, H23 and H727 cells were tested twice in the course of performing the experiments described within this publication (Supplementary Fig. S4). Inhibitors of UPS pathways used in this study were purchased from commercial vendors: carfilzomib (LC Laboratories, Woburn, MA), bortezomib (ChemieTek, Indianapolis, IN), MG-132 (EMD Millipore, San Diego, CA), PYR-41 (ApexBio, Houston, TX), and P5091 (ApexBio, Houston, TX). The following proteasome fluorogenic substrates were used: Suc-LLVY-AMC (Bachem, Torrance, CA; I-1395), Ac-WLA-AMC (Boston Biochem, Cambridge, MA; S-330), Ac-nLPnLD-AMC (Bachem; I-1850), Ac-RLR-AMC (Boston Biochem; S-290), Ac-ANW-AMC (Boston Biochem; S-320), and Ac-PAL-AMC (Boston Biochem; S-310). Human recombinant Interferon-γ was purchased from eBioscience (San Diego, CA).

### Cell viability assay

Cell viability was determined by CellTiter 96 AQueous One Solution Cell Proliferation assay (Promega, Madison, WI) according to the manufacturer’s protocol. Briefly, cells were seeded at a density of 5,000–10,000 per well in 96-well plates and allowed 24 hours to attach. After cells were treated with the indicated concentrations of compounds for 72 hours, cell viability was measured using the reagent provided in the assay kit. Absorbance at 490 nm was measured using a SpectraMax M5 microplate reader (Molecular Devices, Sunnyvale, CA). Results were analyzed using GraphPad Prism (La Jolla, CA).

### Proteasome activity profiling

Subunit-selective fluorogenic peptide substrates were used to measure the catalytic activities of individual catalytic subunits by monitoring the rate of substrate hydrolysis over time. Briefly, protein lysates were prepared using passive lysis buffer (Promega, Madison, WI) and diluted in 20S proteasome assay buffer (20 mM Tris-HCl, 0.5 mM EDTA, 0.035% SDS, pH 8.0). Enzyme reactions were initiated by the addition of proteasome substrates. Substrates and concentrations were used as following: Suc-LLVY-AMC (β5/5i, 100 μM), Ac-WLA-AMC (β5, 20 μM), Ac-nLPnLD-AMC (β1,100 μM), Ac-RLR-AMC (β2/2i, 20 μM), Ac-ANW-AMC (β5i, 100 μM), and Ac-PAL-AMC (β1i activity, 100 μM). Fluorescence signals were measured over 1 hour at one reading per one minute using a SpectraMax M5 microplate reader at the excitation and emission wavelengths of 360 and 460 nm, respectively.

### Immunoblotting analysis

Total cell lysates containing equivalent protein content were separated by 12% SDS-PAGE and transferred to polyvinylidene difluoride membranes (Millipore, Billerica, MA) via a semi-dry apparatus. Membranes were then blocked in 5% non-fat dry milk (Bio-Rad, Hercules, CA) in Tris-buffered saline with 0.05% Tween-20 (TBST) for 1 h at room temperature, followed by incubation with 3% BSA in TBST containing the respective primary antibodies overnight at 4 °C: β1 (Enzo Life Sciences; PW8140), β2 (Enzo Life Sciences; PW8145), β5 (Thermo Scientific; PA1-977), β1i (Abcam; ab3328), β5i (Abcam; ab3329), β-actin (Novus Biologicals; NB600-501), and β2i (Santa Cruz; sc-133236; 3% milk-TBST used for dilution). Membranes were then washed five times with TBST and incubated with HRP (Horse radish peroxidase)-conjugated secondary antibodies for 1 hour at room temperature. Immunoreactive bands were visualized using SuperSignal West Femto Chemiluminescent Substrate (Thermo Scientific, Rockford, IL) and X-ray film (Thermo Scientific or GeneMate).

### IFN-γ-induced transformation of the proteasome into the immunoproteasome

H727 and H23 cells were treated with 150 U⋅ml^−1^ of IFN-γ or vehicle for 24 h. At the end of IFN-γ treatment, the cells were washed with PBS three times and then cultured for an additional 24 hours. Afterwards the cells were sub-cultured into a 96-well plate, and cell viability assays was performed using CellTiter 96 AQueous One Solution Cell Proliferation assay (Promega, Madison, WI) as described above. The remaining cells were then used for immunoblotting analysis and proteasome activity assays.

### Knockdown of proteasome catalytic subunits

Cells were transfected with ON-TARGET Plus Smart Pool siRNAs (Dharmacon, Lafayette, CO) using Lipofectamine 2000 transfection reagent (Invitrogen, Carlsbad, CA), according to the manufacturer’s instructions. H727 cells were plated in a 6-well plate at a density of 5 × 10^5^ cells per well and allowed at least 24 h to attach. Cells were then transfected with 100 nmole of siRNAs and Lipofectamine 2000. At 4 h post-transfection, serum-free Opti-MEM medium (Invitrogen, Carlsbad, CA) was replaced with complete medium and the cells were incubated for 48 h. The following siRNA pools were used: PSMB5 (L-004522-00-0020), PSMB7 (L-006021-00-0020), PSMB8 (L-006022-00-0020), PSMB9 (L-006023-00-0005), and PSMB10 (L-006019-00-0020). For the negative control, human non-targeting scrambled siRNA (D-001810-10) was used.

### Statistics

Results are expressed as means ± S.D. Statistical significance of the observed differences was determined using Student’s t-test (with the Holm-Sidak method when appropriate). All statistical analyses were carried out using GraphPad Prism 7.04 (GraphPad Software).

### Collection of primary MM samples

Cryopreserved MM primary cells isolated from the bone marrow or peripheral blood of patients with no reported history of PI treatment were purchased from Conversant Biologics (Huntsville, AL) and AllCells (Alameda, CA). CD138-positive cells were isolated from patient samples immediately after thawing using human CD138 microbeads (Miltenyi Biotec), whole blood column kit (Miltenyi Biotec), MidiMACS magnetic separator (Miltenyi Biotec), and 30 µm MACS SmartStrainers (Miltenyi Biotec). Purified cells were plated on white 96-well cell culture plates at 40,000 cells per well in RPMI 1640 media supplemented with 10% FBS. Cells were treated with proteasome inhibitors for 48 hours before viability assessment via CellTiter-Glo Luminescent Cell Viability Assay (Bio-Rad).

## Supplementary information

H727 cells are inherently resistant to the proteasome inhibitor carfilzomib, yet require proteasome activity for cell survival and growth

## Data Availability

The data that support the findings of this study are available from the corresponding author upon reasonable request.
