# Supplementary material for: H727 cells are inherently resistant to the proteasome inhibitor carfilzomib, yet require proteasome activity for cell survival and growth
Source: Sci Rep. 2019 Mar 11;9:4089. doi: 10.1038/s41598-019-40635-1 (PMC6411724; doi:10.1038/s41598-019-40635-1)
Supplement: Supplementary file 1 — H727 cells are inherently resistant to the proteasome inhibitor carfilzomib, yet require proteasome activity for cell survival and growth [file 41598_2019_40635_MOESM1_ESM.pdf]

H727 cells are inherently resistant to the proteasome inhibitor carfilzomib, yet require proteasome activity for cell survival and growth

Min Jae Lee,[1] Zachary Miller,[1] Ji Eun Park,[2] Deepak Bhattarai,[1] Wooin Lee,[2] Kyung Bo Kim[1,*]

[1]Department of Pharmaceutical Sciences, University of Kentucky, Lexington KY; [2]College of Pharmacy and Research Institute of Pharmaceutical Sciences, Seoul National University, Seoul, Korea

Contents

S-1 Title Page

S-2 Supplemental Figure 1. Sequencing analysis of the *PSMB5* (encoding β5) and *PSMB8* (encoding β5i) genes in H727 cells

S-6 Supplemental Figure 2. Immunoblots indicating expression of both cP and iP catalytic subunits in H727, H23, Panc-1, and RPMI 8226 cells

S-6 Supplemental Figure 3. Effects of siRNA knockdown of β5 on Btz sensitivity in H727 cells

S-7 Supplemental Figure 4. Mycoplasma contamination test in H727 and H23 cells

Supplemental Figure 1. Sequencing analysis of the *PSMB5* (encoding β5) and *PSMB8* (encoding β5i) genes in H727 cells

Sequencing of PSMB5 and PSMB8 (PSMB8E2, isoform E2) open-reading frames (ORFs) in H727 and H23 (control) cells was performed to identify any possible mutations. For each cell line RNA was extracted from approximately 5 million cells growing on a 150 mm dish using Invitrogen's PureLink RNA Mini Kit. Following elution and spectrophotometric quantification, 500 ng of total RNA per cell line were used for cDNA synthesis using Invitrogen's SuperScript III First-Strand Synthesis System with oligo$(dT)_{20}$ primers. H727 and H23 cDNA were used in 30-cycle conventional PCR reactions with primer pairs covering the PSMB5 and PSMB8E2 ORFs. A total of 50 ng RNA-equivalent cDNA was used per 50 uL PCR reaction using Invitrogen Phusion Hot Start II DNA polymerase. Completed PCR reactions were loaded onto 1% agarose gel and subjected to electrophoresis. For each reaction a major band (>80% total) of the expected size was identified and cut from the gel. The PCR product was isolated from the gel slice using a silica column-type gel extraction kit. After spectrophotometric quantification of the eluted DNA, an appropriate amount of each PCR product was pre-mixed with a forward primer (PSMB5) or in separate reactions with either forward or reverse primer (PSMB8E2) and submitted to Eurofins Genomics for Sanger sequencing. H23 and H727 sequences in .seq format were compared to the appropriate reference sequence in Clustal Omega. For PSMB8E2 reverse sequencing reads, sequences were compared to the reverse compliment of the NCBI reference sequence. Output from Clustal Omega was sent to JalView 2.10.5 for visualization. Bases with 100% consensus between H23, H727, and the reference sequence are highlighted. No mutations were detected for H23 or H727 in the PSMB5 or PSMB8E2 coding sequences relative to the published reference sequence.

PSMB5 NCBI RefSeq Accession Number: NM_002797.4
PSMB5 Primers:

Forward: 5'-AAT AGG AAG TGA AGC TGT GAC G-3'
Reverse: 5'-GTG TCC GTA TTA CCA ATG ACA GT-3'

PSMB8E2 NCBI RefSeq Accession Number: NM_148919.3
PSMB8E2 Primers:

Forward: 5'-AGC GGA CAG ATC TCT GGG TG-3'
Reverse: 5'-TTA ACG TGG CTT AGG TCC CTG-3'

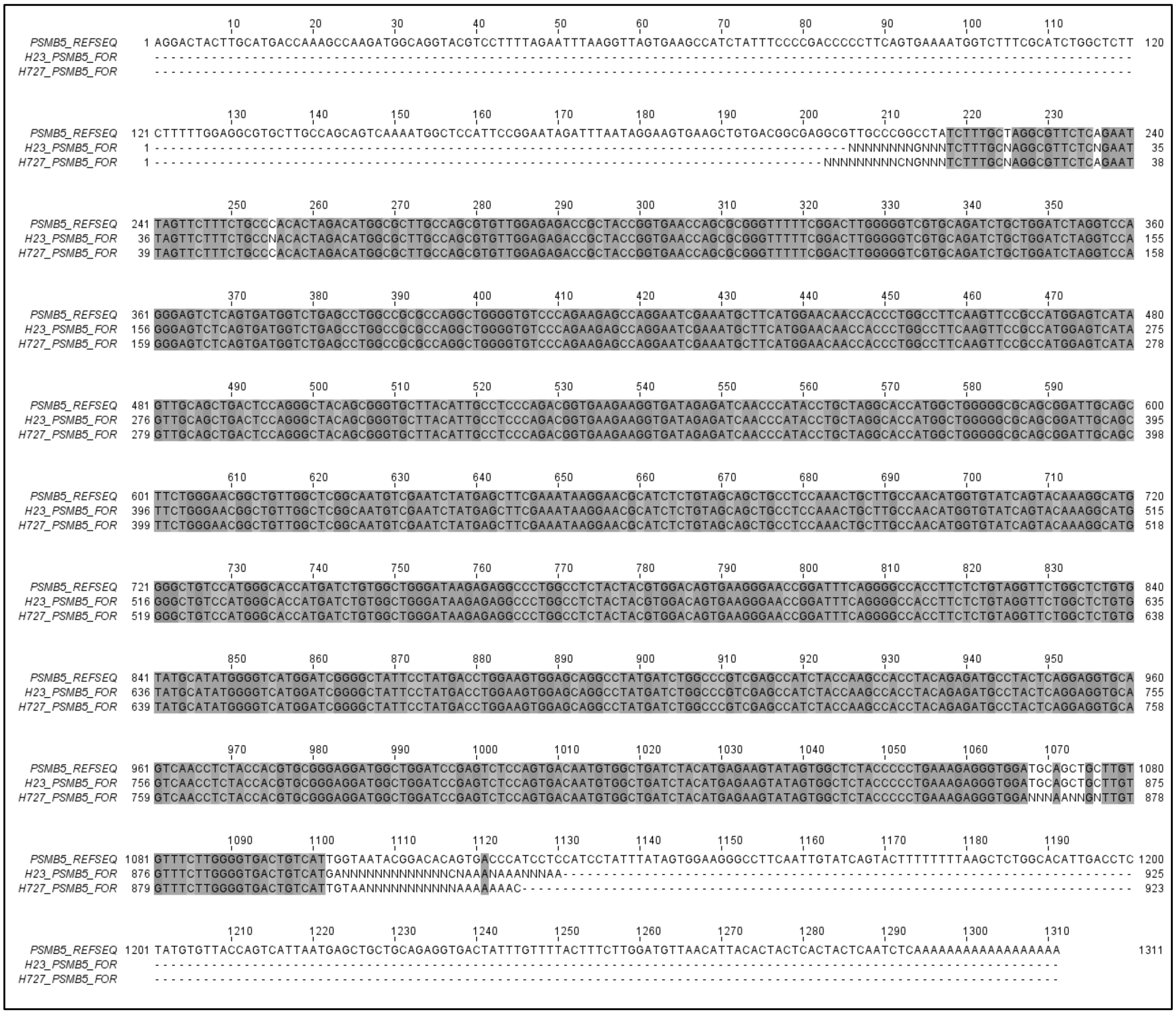

Supplemental Figure 1-1. Alignment of H23 and H727 PSMB5 forward sequencing reads to the PSMB5 reference sequence. The PSMB5 coding sequence is located at 265-1056 and no mutation was detected in both H23 and H727 cells.

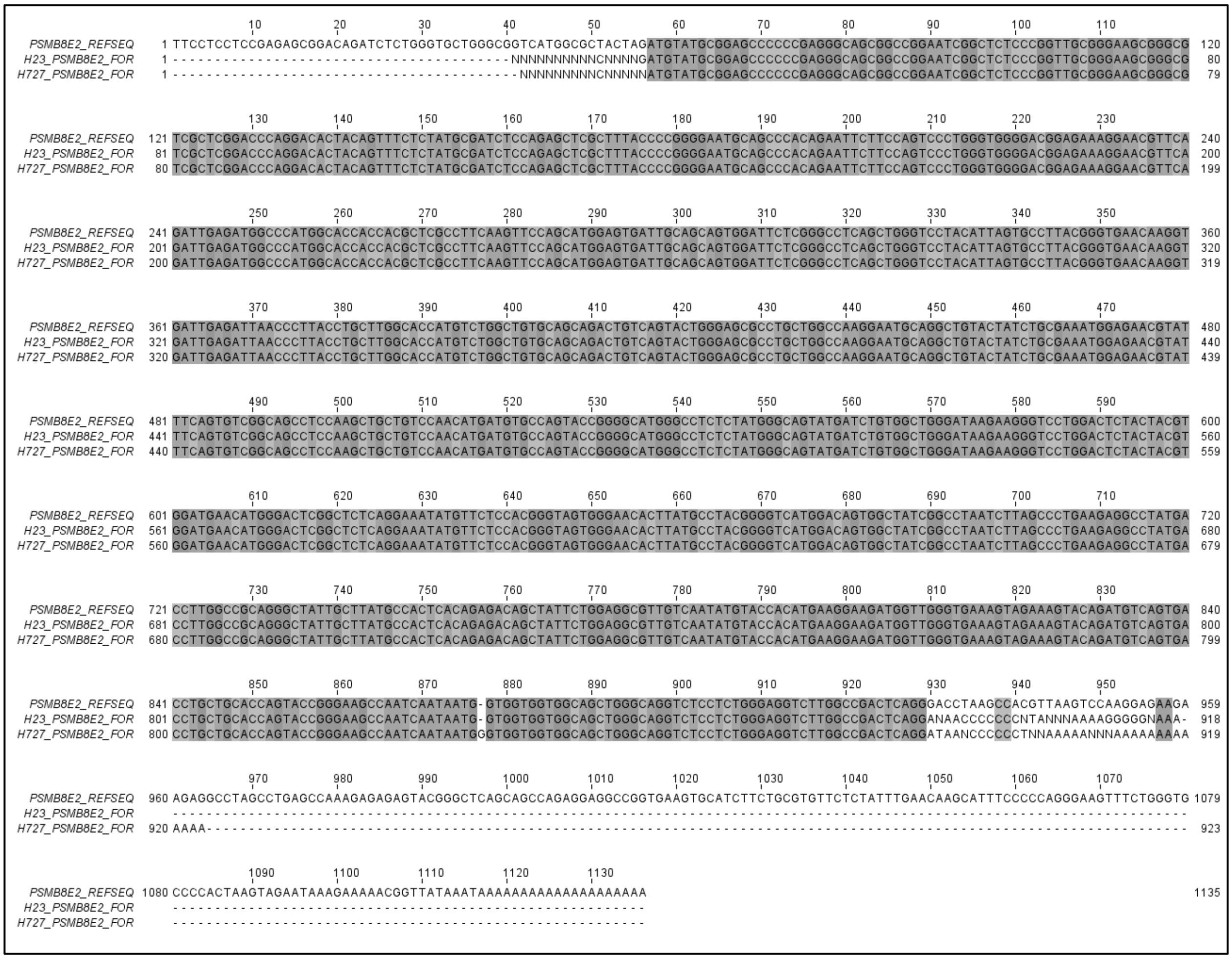

Supplemental Figure 1-2. Alignment of H23 and H727 PSMB8E2 forward sequencing reads to the PSMB8E2 reference sequence. The PSMB8 coding sequence is located at 44-874 and no mutation was detected in both H23 and H727 cells.

```
                            cov    pid    1 [         .         .         .         .         :         .         .         .         .         1 100
1 PSMB8E2_REFSEQ_REVCOMP 100.0% 100.0%      TTTTTTTTTTTTTTTTTTTTTATTTATAACCGTTTTTCTTTATTCTACTTAGTGGGGCACCCAGAAACTTCCCTGGGGAAATGCTTGTTCAAATAGAGAA
2 H23_PSMB8E2_REV         80.2%  96.3%      ----------------------------------------------------------------------------------------------------
3 H727_PSMB8E2_REV        80.4%  95.6%      ----------------------------------------------------------------------------------------------------

                            cov    pid  101           .         .         .         .         :         .         .         .         .         2 200
1 PSMB8E2_REFSEQ_REVCOMP 100.0% 100.0%      CACGCAGAAGATGCACTTCACCGGCCTCCTCTGGCTGCTGAGCCCGTACTCTCTCTTTGGCTCAGGCTAGGCCTCTTCTTCTCCTTGGACTTAACGTGGC
2 H23_PSMB8E2_REV         80.2%  96.3%      ----------------------------------------------------------------------------------------------------
3 H727_PSMB8E2_REV        80.4%  95.6%      ----------------------------------------------------------------------------------------------------

                            cov    pid  201           .         .         .         .         :         .         .         .         .         3 300
1 PSMB8E2_REFSEQ_REVCOMP 100.0% 100.0%      TTAGGTCCCTGAGTCGGCCAAGACCTCCCAGAGGAGACCTGCCCAGCTGCCACCACCACCATTATTGATTGGCTTCCCGGTACTGGTGCAGCAGGTCACT
2 H23_PSMB8E2_REV         80.2%  96.3%      -----------------------NNNNNNNNNAGNNANCTGCCCAGCTGCCACCACCACCATTATTGATTGGCTTCCCGGTACTGGTGCAGCAGGTCACT
3 H727_PSMB8E2_REV        80.4%  95.6%      ---------------------NNNNNNCNNNNNNNNANCTGCCCAGCTGCCACCACCACCATTATTGATTGGCTTCCCGGTACTGGTGCAGCAGGTCACT

                            cov    pid  301           .         .         .         .         :         .         .         .         .         4 400
1 PSMB8E2_REFSEQ_REVCOMP 100.0% 100.0%      GACATCTGTACTTTCTACTTTCACCCAACCATCTTCCTTCATGTGGTACATATTGACAACGCCTCCAGAATAGCTGTCTCTGTGAGTGGCATAAGCAATA
2 H23_PSMB8E2_REV         80.2%  96.3%      GACATCTGTACTTTCTACTTTCACCCAACCATCTTCCTTCATGTGGTACATATTGACAACGCCTCCAGAATAGCTGTCTCTGTGAGTGGCATAAGCAATA
3 H727_PSMB8E2_REV        80.4%  95.6%      GACATCTGTACTTTCTACTTTCACCCAACCATCTTCCTTCATGTGGTACATATTGACAACGCCTCCAGAATAGCTGTCTCTGTGAGTGGCATAAGCAATA

                            cov    pid  401           .         .         .         .         :         .         .         .         .         5 500
1 PSMB8E2_REFSEQ_REVCOMP 100.0% 100.0%      GCCCTGCGGCCAAGGTCATAGGCCTCTTCAGGGCTAAGATTAGGCCGATAGCCACTGTCCATGACCCCGTAGGCATAAGTGTTCCCACTACCCGTGGAGA
2 H23_PSMB8E2_REV         80.2%  96.3%      GCCCTGCGGCCAAGGTCATAGGCCTCTTCAGGGCTAAGATTAGGCCGATAGCCACTGTCCATGACCCCGTAGGCATAAGTGTTCCCACTACCCGTGGAGA
3 H727_PSMB8E2_REV        80.4%  95.6%      GCCCTGCGGCCAAGGTCATAGGCCTCTTCAGGGCTAAGATTAGGCCGATAGCCACTGTCCATGACCCCGTAGGCATAAGTGTTCCCACTACCCGTGGAGA

                            cov    pid  501           .         .         .         .         :         .         .         .         .         6 600
1 PSMB8E2_REFSEQ_REVCOMP 100.0% 100.0%      ACATATTTCCTGAGAGCCGAGTCCCATGTTCATCCACGTAGTAGAGTCCAGGACCCTTCTTATCCCAGCCACAGATCATACTGCCCATAGAGAGGCCCAT
2 H23_PSMB8E2_REV         80.2%  96.3%      ACATATTTCCTGAGAGCCGAGTCCCATGTTCATCCACGTAGTAGAGTCCAGGACCCTTCTTATCCCAGCCACAGATCATACTGCCCATAGAGAGGCCCAT
3 H727_PSMB8E2_REV        80.4%  95.6%      ACATATTTCCTGAGAGCCGAGTCCCATGTTCATCCACGTAGTAGAGTCCAGGACCCTTCTTATCCCAGCCACAGATCATACTGCCCATAGAGAGGCCCAT

                            cov    pid  601           .         .         .         .         :         .         .         .         .         7 700
1 PSMB8E2_REFSEQ_REVCOMP 100.0% 100.0%      GCCCCGGTACTGGCACATCATGTTGGACAGCAGCTTGGAGGCTGCCGACACTGAAATACGTTCTCCATTTCGCAGATAGTACAGCCTGCATTCCTTGGCC
2 H23_PSMB8E2_REV         80.2%  96.3%      GCCCCGGTACTGGCACATCATGTTGGACAGCAGCTTGGAGGCTGCCGACACTGAAATACGTTCTCCATTTCGCAGATAGTACAGCCTGCATTCCTTGGCC
3 H727_PSMB8E2_REV        80.4%  95.6%      GCCCCGGTACTGGCACATCATGTTGGACAGCAGCTTGGAGGCTGCCGACACTGAAATACGTTCTCCATTTCGCAGATAGTACAGCCTGCATTCCTTGGCC

                            cov    pid  701           .         .         .         .         :         .         .         .         .         8 800
1 PSMB8E2_REFSEQ_REVCOMP 100.0% 100.0%      AGCAGGCGCTCCCAGTACTGACAGTCTGCTGCACAGCCAGACATGGTGCCAAGCAGGTAAGGGTTAATCTCAATCACCTTGTTCACCCGTAAGGCACTAA
2 H23_PSMB8E2_REV         80.2%  96.3%      AGCAGGCGCTCCCAGTACTGACAGTCTGCTGCACAGCCAGACATGGTGCCAAGCAGGTAAGGGTTAATCTCAATCACCTTGTTCACCCGTAAGGCACTAA
3 H727_PSMB8E2_REV        80.4%  95.6%      AGCAGGCGCTCCCAGTACTGACAGTCTGCTGCACAGCCAGACATGGTGCCAAGCAGGTAAGGGTTAATCTCAATCACCTTGTTCACCCGTAAGGCACTAA

                            cov    pid  801           .         .         .         .         :         .         .         .         .         9 900
1 PSMB8E2_REFSEQ_REVCOMP 100.0% 100.0%      TGTAGGACCCAGCTGAGGCCCGAGAATCCACTGCTGCAATCACTCCATGCTGGAACTTGAAGGCGAGCGTGGTGGTGCCATGGGCCATCTCAATCTGAAC
2 H23_PSMB8E2_REV         80.2%  96.3%      TGTAGGACCCAGCTGAGGCCCGAGAATCCACTGCTGCAATCACTCCATGCTGGAACTTGAAGGCGAGCGTGGTGGTGCCATGGGCCATCTCAATCTGAAC
3 H727_PSMB8E2_REV        80.4%  95.6%      TGTAGGACCCAGCTGAGGCCCGAGAATCCACTGCTGCAATCACTCCATGCTGGAACTTGAAGGCGAGCGTGGTGGTGCCATGGGCCATCTCAATCTGAAC

                            cov    pid  901           .         .         .         .         :         .         .         .         .         0 1000
1 PSMB8E2_REFSEQ_REVCOMP 100.0% 100.0%      GTTCCTTTCTCCGTCCCCACCCAGGGACTGGAAGAATTCTGTGGGCTGCATTCCCCGGGGTAAAGCGAGCTCTGGAGATCGCATAGAGAAACTGTAGTGT
2 H23_PSMB8E2_REV         80.2%  96.3%      GTTCCTTTCTCCGTCCCCACCCAGGGACTGGAAGAATTCTGTGGGCTGCATTCCCCGGGGTAAAGCGAGCTCTGGAGATCGCATAGAGAAACTGTAGTGT
3 H727_PSMB8E2_REV        80.4%  95.6%      GTTCCTTTCTCCGTCCCCACCCAGGGACTGGAAGAATTCTGTGGGCTGCATTCCCCGGGGTAAAGCGAGCTCTGGAGATCGCATAGAGAAACTGTAGTGT

                            cov    pid 1001           .         .         .         .         :         .         .         .         .         1 1100
1 PSMB8E2_REFSEQ_REVCOMP 100.0% 100.0%      CCTGGGTCCGAGCGACGCCCGCTTCCCGCAACCGGGAGAGCCGATTCCGGCCGCTGCCCTCGGGGGGCTCCGCATACATCTAGTAGCGCCATGACCGCCC
2 H23_PSMB8E2_REV         80.2%  96.3%      CCTGGGTCCGAGCGACGCCCGCTTCCCGCAACCGGGAGAGCCGATTCCGGCCGCTGCCCTCGGGGGGCTCCGCATACATCTAGTAGCGCCATGACCGCCC
3 H727_PSMB8E2_REV        80.4%  95.6%      CCTGGGTCCGAGCGACGCCCGCTTCCCGCAACCGGGAGAGCCGATTCCGGCCGCTGCCCTCGGGGGGCTCCGCATACATCTAGTAGCGCCATGACCGCCC

                            cov    pid 1101           .         .         .    ] 1135
1 PSMB8E2_REFSEQ_REVCOMP 100.0% 100.0%      AGCACCCAGAGATCTGTCCGCTCTCGGAGGAGGAA
2 H23_PSMB8E2_REV         80.2%  96.3%      AGCACCANNNNTTTTNNCCCCNNNAANNNAA--AA
3 H727_PSMB8E2_REV        80.4%  95.6%      AGCACCANNNNNNNNNNCNNNNNAAANNAAA--AA
```

Supplemental Figure 1-3. Alignment of H23 and H727 PSMB8E2 reverse sequencing reads to the PSMB8E2 reference sequence reverse compliment. The PSMB8 protein coding sequence reverse compliment is located at 262-1092 and is fully covered by both sequencing reads.

Supplemental Figure 2. Immunoblots showing differential expression of proteasome catalytic subunits in H727, H23, Panc-1, and RPMI 8226 cells

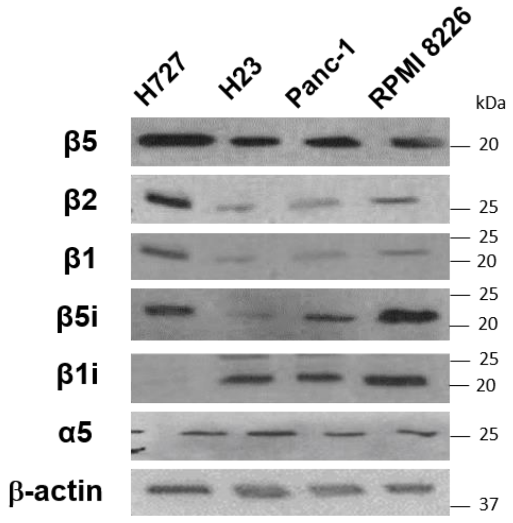

Supplemental Figure 3. Effects of siRNA knockdown of β5 on Btz sensitivity in H727 cells

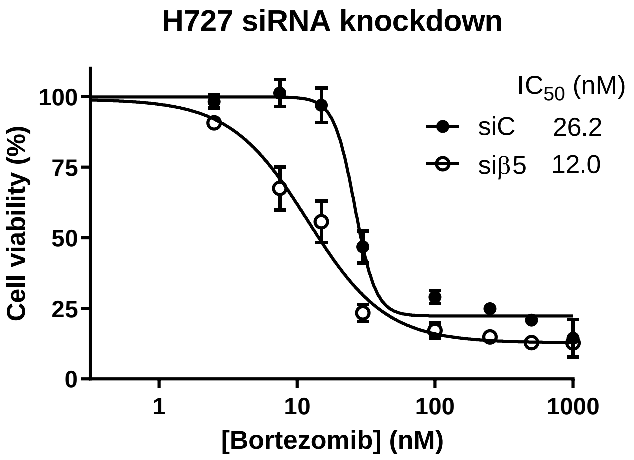

Supplemental Figure 4. Mycoplasma contamination test in H727 and H23 cells

Southern Biotech Mycoplasma Detection Kit catalogue number 13100-01 was purchased and used according to the manufacturer's instructions. This kit amplifies the 16S rRNA coding region of *Mycoplasma* genomic DNA present in *Mycoplasma*-contaminated cell culture supernatants. Invitrogen Platnium Taq polymerase was purchased separately. Mycoplasma testing of cell culture supernatants from H23 and H727 cells was performed on two separate occasions. Each reaction included an internal control to detect PCR inhibition (270 bp product). PCR reactions were analyzed via electrophoresis in 1% agarose gel stained with ethidium bromide. A negative control reaction and a positive control reaction were included for each gel. Invitrogen 1 Kb Plus ladder was used to identify approximate band sizes.

Representative gel image:

Lanes 1-4,5-11: Various cell lines, *Mycoplasma* negative
Lane 5: Mycoplasma-infected cell lin econtrol.
Lane 12: H727 cell line, *Mycoplasma* negative
Lane 13: H23 cell line, *Mycoplasma* negative
Lane 14: Negative control reaction (distilled water)
Lane 15: Positive control reaction (*M. orale*, 503 bp amplicon)

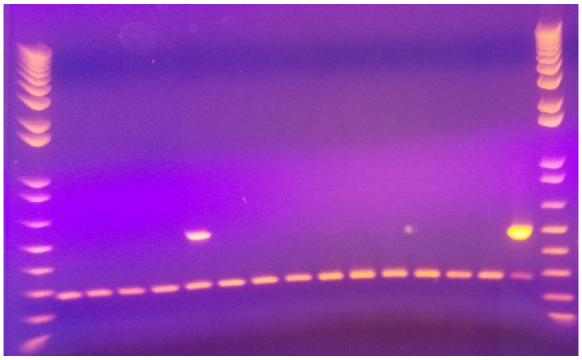

1 2 3 4 5 6 7 8 9 10 11 12 13 14 15
